# A dataset from the Cryptogamia-Lichenes section of the Herbarium Universitatis Taurinensis (TO)

**DOI:** 10.3897/BDJ.12.e134717

**Published:** 2025-02-06

**Authors:** Rosanna Piervittori, Deborah Isocrono, Enrica Matteucci, Mariagrazia Morando, Luca Dessì, Laura Guglielmone, Heimo Rainer, Stefano Martellos, Andrea Moro, Pier Luigi Nimis, Matteo Conti, Sergio Enrico Favero-Longo

**Affiliations:** 1 Department of Life Sciences and Systems Biology, University of Torino, Viale Mattioli 25, I-10125, Torino, Italy Department of Life Sciences and Systems Biology, University of Torino, Viale Mattioli 25, I-10125 Torino Italy; 2 Department of Agricultural, Forest and Food Sciences (DISAFA), University of Torino, Largo Paolo Braccini 2, I-10095 Grugliasco, Italy Department of Agricultural, Forest and Food Sciences (DISAFA) University of Torino, Largo Paolo Braccini 2, I-10095 Grugliasco Italy; 3 Dipartimento per lo Sviluppo Sostenibile e la Transizione Ecologica dell'Università del Piemonte Orientale, Vercelli, Italy Dipartimento per lo Sviluppo Sostenibile e la Transizione Ecologica dell'Università del Piemonte Orientale Vercelli Italy; 4 Department of Botany, Natural History Museum Vienna, Burgring 7, A-1010, Vienna, Austria Department of Botany, Natural History Museum Vienna, Burgring 7, A-1010 Vienna Austria; 5 Department of Life Sciences, University of Trieste, Via Giorgieri 10, I-34127, Trieste, Italy Department of Life Sciences, University of Trieste, Via Giorgieri 10, I-34127 Trieste Italy; 6 Centro Interuniversitario per le Biodiversità Vegetale Big Data - PLANT DATA, Department of Biological, Geological and Environmental Sciences, Alma Mater Studiorum University of Bologna, Bologna, Italy Centro Interuniversitario per le Biodiversità Vegetale Big Data - PLANT DATA, Department of Biological, Geological and Environmental Sciences, Alma Mater Studiorum University of Bologna Bologna Italy

**Keywords:** collection, biodiversity, georeference, Italy, lichens

## Abstract

**Background:**

The section Cryptogamia-Lichenes of the Herbarium Universitatis Taurinensis (TO) includes ca. 34,600 lichen specimens, organised in the historical (ca. 30,700 specimens, mostly from the 19^th^ century) and modern (ca. 3,900 specimens collected from 1978, out of which ca. 3400 from Italy) collections. Specimens from the administrative regions of Piemonte and Valle d’Aosta (NW Italy) are the core of the modern collection, documenting floristic and vegetation studies, as well as biomonitoring campaigns and investigations on the biodeterioration of the stone cultural heritage.

**New information:**

The dataset of the Italian materials of the modern lichenological collection of TO, with 3,365 samples, is fully georeferenced and accessible in the Global Biodiversity Information Facility (GBIF), in the Jointly Administered Herbarium Management System and Specimen Database (JACQ) and in the Information System of Italian Lichens (ITALIC). With regard to the historical collection, only a set of 59 recently revised specimens is available on the mentioned platforms, but most of the materials are accessible as digital images on the website of the project HERB-TO-CHANGE.

## Introduction

Since lichens are sensitive to several environmental parameters, such as climatic factors and airborne chemicals, they can be adopted as bioindicators of the effects of climate change and land use, as well as of air pollution and other human impacts (Giordani and Brunialti 2015, Giordani 2019). Historical records are a mandatory reference for identifying trends in lichen diversity as well as for interpreting potential driving factors (Nelsen and Lumbsch 2020). Herbarium collections are recognised as fundamental archives of spatial and temporal data on lichen distribution, as well as material sources for manipulative and analytical investigations, supporting studies from local to global environmental changes (e.g. Farkas et al. (2022), Wu et al. (2023)). Accordingly, accessibility to herbarium data is increasingly advocated and promoted through several gateways. As far as Italy is concerned, a remarkable advancement was produced thanks to the aggregation of data from several modern Italian lichen herbaria into ITALIC, the information system on Italian lichens (Martellos et al. 2023). The system currently aggregates more than 88,000 records from 13 herbaria, amongst which the datasets from the Herbarium Universitatis Tergestinae (TSB, Conti et al. 2023) and the herbarium of the Botanical Garden of the University of Calabria (CLU, Conti et al. 2024) have already been published in GBIF.

A dataset from the Cryptogamia-Lichenes section of the Herbarium Universitatis Taurinensis (TO), encompassing the whole modern collection and records from the historical collection revised by experts in the last decades, was recently published both in the JACQ platform (in the framework of the project "HERB-TO-CHANGE" dedicated to the digitisation of TO) and in the GBIF (thanks to the support of the project Dryades, Nimis et al. (2003)).

The cryptogamic sections of TO include over 130,000 specimens, of which approx. 34,600 are lichens (Piervittori and Pistarino 1990, Isocrono et al. 2017). The historical lichenological collection (Collezione storica) consists of ca. 30,700 specimens. It is the product of a reorganisation that occurred at the end of the 19^th^ century, during which specimens from different herbaria were merged and sorted systematically and alphabetically. The historical collection includes specimens prepared by some of the most relevant Italian lichenologists of the 19^th^ century (e.g. Anzi, Baglietto, Carestia and De Notaris), as well as those from several series of exsiccata (e.g. Philipp Hepp’s "Die Flechten Europas", G. W. Koerber’s "Lichenes selecti Germanici", Leighton’s "Lichenes britannici exsiccati" and the Rabenhorst’s collections). Additionally, some hundreds of historical specimens are preserved in a few distinct closed collections (e.g. Terraneo, Moris and Hill). While actions are underway to digitise the historical materials, they are far from complete, mostly due to the need for revision work, together with issues related to outdated nomenclature or to labels referring to paper inventories which are hard to retrieve. Nevertheless, most of the sheets from the historical collection were recently made accessible as digital images on the website of the project "HERB-TO-CHANGE".

The modern lichenological collection (Collezione attuale), started in 1978 by Rosanna Piervittori and is still being implemented, includes approx. 3,900 specimens, out of which 3,365 are from Italy, mostly resulting from field research activities carried out by the Laboratory of Lichenology of the University of Torino (LabLich-UniTO). Specimens collected in the NW Italian Alps are the core of the collection, together with those documenting biomonitoring campaigns in the Po Plain and lichen diversity surveys in cultural heritage sites. The collection also includes specimens collected during field activities of the Italian Lichen Society. All the Italian modern specimens were digitised and the related dataset is now accessible in ITALIC (Nimis 2024, GBIF 2024, JACQ 2024).

## Sampling methods

### Study extent

The digitised dataset of the Cryptogamia-Lichenes section of TO includes all the Italian materials of the modern collection (n = 3,365) and a set of recently revised Italian specimens of the historical collection (n = 59).

### Sampling description

The modern lichenological collection is mostly made of specimens gathered during field research activities of LabLich-UniTO (ISO 9001:2015), which include floristic surveys, vegetation relevés, biomonitoring campaigns and investigations dedicated to the biodeterioration of the stone cultural heritage. Specimens mostly consist of whole thalli, with the exception of samples from heritage surfaces, from which only thallus fragments were usually collected because of sampling limitations for conservation reasons. These small samples are preserved in tubes, while whole thallus specimens and, when present, their substrates are wrapped in soft paper, according to Obermayer (2002). All specimens are then stored in paper envelopes (approx. 12 × 10 cm), organised within three series of boxes (45 × 15 × 10 cm), each devoted to a distinct geographic area, namely Piemonte (PIE), Valle d’Aosta (AO) and the rest of the world (HUT). Each envelope can host more specimens if these thalli grow together on the substrate, particularly in the case of saxicolous lichens. Envelopes have labels that report the identification of the specimen(s), the locality and date of the collection, the collector(s) and ecological notes (substrate, habitat). A progressive ID is assigned to each specimen (from 1 to 3852 on 16-02-2023); a distinct numbering is used to identify each envelope (from 1 to 2669 on 16-02-2023). As part of the HERB-TO-CHANGE project, each specimen is also identified with a number and a QR code generated by the JACQ platform. Both are printed on a label that is fixed to the envelope.

Alternatively, the specimens of the historical collection were mounted using the same method used for phanerogamic collections in the 19^th^ century. Lichen thalli themselves, or the envelopes that contain them, were glued or stapled on to sheets of paper (approx. 30 × 46 cm) also used for vascular plants exsiccata. Identification and collection data were hand- or typewritten on the envelopes or on cards. In some cases, cuts of printed inventories were also used. Recently revised specimens (n = 159) are still preserved on the sheets, which are stored in distinct folders for each taxon (4,747 folders, alphabetically organised in 143 groups to manage their arrangement in the dedicated closets, compose the whole historical collection). All the revised specimens of the historical collection are identified by a number and a QR code generated by the JACQ platform.

The label metadata of all the specimens were digitised in a spreadsheet and then imported into a MySQL database to be aggregated into ITALIC, GBIF and JACQ platforms. Images of each envelope and its content were acquired using a scanner ScanSnap SV600 (FujiTsu) at a 600 dpi resolution and have been made available on the JACQ platform.

### Quality control

The collection and identification of the modern materials were carried out by experienced lichenologists of LabLich-UniTO (Piervittori R., Isocrono D., Matteucci E., Favero-Longo S.E.). Italian and foreign specialists contributed to the revision of specimens from the historical collection and of certain taxonomic groups from the modern collection. For each specimen, both the first identification and the currently accepted name (according to Nimis (2016)), were reported in the dataset. For several specimens collected during field surveys in the 2000s, the coordinates of the collecting site were acquired using GPS devices (Garmin 12, Garmin eTrex Summit). In other cases, post-hoc georeferencing was carried out by means of regional GIS maps, Google Maps and Google Earth, following the best practices by Chapman and Wieczorek (2020).

## Geographic coverage

### Description

**Figure 1.**
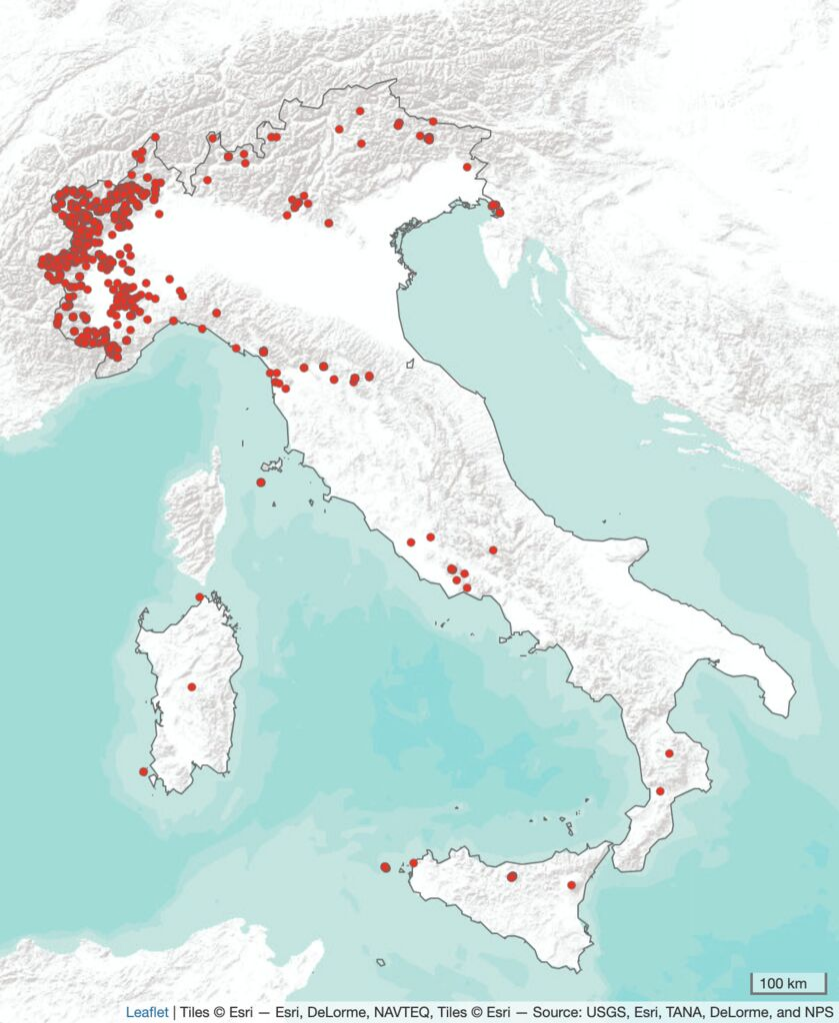
Distribution map of the specimens included in the database, created with tmap (Tennekes 2018).

All the specimens were collected in Italy (Fig. 1), mostly in the administrative regions of Piemonte (n = 1,610; 47%) and Valle d’Aosta (n = 1,547; 45%). A small set of specimens were collected in other parts of Italy (Toscana, n = 74; Friuli Venezia Giulia, 53; Sicilia, 44; Lombardia, 44; Liguria, 24; Lazio, 10; Veneto, 8; Sardegna, 4; Trentino Alto Adige, 3; Calabria, 2; Abruzzo, 1).

### Coordinates

37.837 and 46.744 Latitude; 6.664 and 14.062 Longitude.

## Taxonomic coverage

### Description

According to the checklist of the lichens of Italy (Nimis 2016), the materials of the dataset belong to 601 species, 209 genera, 64 families, 28 orders and 8 classes.

The most represented families and genera are shown in Table 1 and Table 2, respectively. The distribution of taxa and specimens amongst kingdoms, phyla, classes, orders, families and genera can be graphically visualised as a Krona graph (Ondov et al. 2011), an interactive multi-layered pie chart (Suppl. material 1) or in tabular format (Suppl. material 2).

## Temporal coverage

### Notes

**Figure 2.**
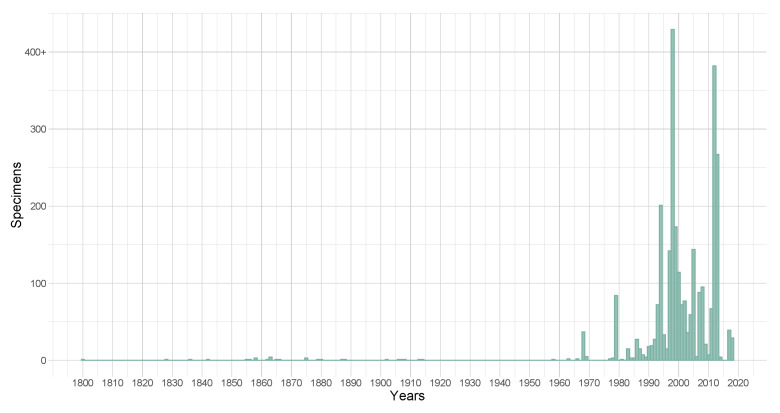
Specimens collected per year.

Specimens in the database were collected between 1800 and 2018 (Fig. 2). The specimens from the historical collection date from 1830 to 1936. The specimens from the modern collection include those collected from 1978 to 2018 and 45 specimens collected between 1958 and 1977 by Franco Montacchini, Rosanna Piervittori’s supervisor.

## Usage licence

### Usage licence

Other

### IP rights notes

This work is licensed under a Creative Commons Attribution Non-Commercial (CC-BY-NC 4.0) Licence.

## Data resources

### Data package title

Herbarium TO / Cryptogamia-Lichenes

### Resource link

https://doi.org/10.15468/5vtp55

### Alternative identifiers

https://cloud.gbif.org/eca/resource?r=to_cryptogamia-lichenes

### Number of data sets

1

### Data set 1.

#### Data set name

Herbarium TO / Cryptogamia-Lichenes

#### Data format

Darwin Core

#### Download URL

https://cloud.gbif.org/eca/archive.do?r=to_cryptogamia-lichenes

#### Description

The section Cryptogamia-Lichenes includes ca. 30,700 samples collected from the second half of the 18^th^ century to ca. 1936 (historical section) and ca. 3,900 samples collected and organised from 1978 (modern section) (Favero Longo 2024). The historical section (not digitised yet) includes collections of some important Italian lichenologists of the "golden period" (e.g. Anzi, Baglietto, Carestia, De Notaris etc.) and from European herbaria. Samples collected in Piemonte and Valle d'Aosta, in the framework of the research activities of Rosanna Piervittori's group, are the core of the modern section, documenting the richness of lichen diversity in the NW Alps of Italy.

**Data set 1.** 

Column label	Column description
occurrenceID	A unique identifier for the occurrence.
institutionID	Global Registry of Scientific Collections identifier for the institution.
institutionCode	Acronym in use by the institution having custody of the object.
collectionID	Global Registry of Scientific Collections identifier for the collection.
collectionCode	Acronym identifying the collection from which the record was derived.
basisOfRecord	The specific nature of the data (PreservedSpecimen for all records).
catalogNumber	Identifier for the record within the dataset or collection.
recordedBy	Person or group that collected the specimen.
identifiedBy	Person who identified the specimen.
eventDate	Date in which the specimen was collected.
continent	Continent where the specimen was collected.
country	Country where the specimen was collected.
countryCode	Standardised code for the country.
stateProvince	Administrative region where the specimen was collected.
locality	Description of the place where the specimen was taken.
minimumElevationInMetres	Minimum elevation (in metres) at which the occurrence was recorded.
maximumElevationInMetres	Maximum elevation (in metres) at which the occurrence was recorded.
decimalLatitude	Latitude of the occurrence in decimal degrees.
decimalLongitude	Longitude of the occurrence in decimal degrees.
geodeticDatum	Geodetic datum of the geographic coordinates.
coordinateUncertaintyInMetres	Uncertainty (in metres) associated with the geographic coordinates.
scientificName	Scientific name, with authorship. Aligned to the Italian checklist of lichens.
verbatimIdentification	The taxonomic identification as written on the specimen’s label.
kingdom	Kingdom in which the taxon is classified.
taxonRank	Taxonomic rank of the most specific name.
licence	Terms under which the dataset is made available.
type	The nature of the resource (PhysicalObject for all records).
language	The language of the resource.

## Supplementary Material

3EBB40D5-1185-5EAE-9853-12EC84E87939 10.3897/BDJ.12.e134717.suppl1 Supplementary material 1 Krona graph Data type HTML file Brief description Krona graph of specimens and taxa in the dataset. File: oo_1168471.html https://binary.pensoft.net/file/1168471 Matteo Conti

80FE508B-B6EA-54F4-A0EB-CE883BC00186 10.3897/BDJ.12.e134717.suppl2 Supplementary material 2 Table of taxa and the number of specimens in the Herbarium Data type Table Brief description Table of taxa and the number of specimens in the Herbarium TO / Cryptogamia-Lichenes collection. File: oo_1168472.tsv https://binary.pensoft.net/file/1168472 Matteo Conti

## Figures and Tables

**Table 1.** Families with the highest number of specimens and number of taxa per family in the dataset.

Family	Number of specimens	Number of taxa
Parmeliaceae Zenker	1002	89
Lecanoraceae Körb.	366	54
Physciaceae Zahlbr.	242	45
Teloschistaceae Zahlbr.	187	42
Cladoniaceae Zenker	172	43
Rhizocarpaceae M.Choisy & Hafellner	159	13
Lecideaceae Chevall.	146	27
Umbilicariaceae Chevall.	140	16
Candelariaceae Hakul.	140	9
Peltigeraceae Dumort.	126	20

**Table 2.** Genera with the highest number of specimens and number of taxa per genus in the dataset.

Genus	Number of specimens	Number of taxa
*Lecanora* Ach.	192	28
*Xanthoparmelia* (Vain.) Hale	185	14
*Cladonia* P. Browne	172	43
*Rhizocarpon* DC.	159	13
*Umbilicaria* Hoffm.	140	16
*Physcia* (Schreb.) Michx.	112	15
*Peltigera* Willd.	107	17
*Candelariella* Müll. Arg.	106	8
*Parmelia* Ach.	95	5
*Cetraria* Ach.	93	7
