# Supplementary material for: A dataset from the Cryptogamia-Lichenes section of the Herbarium Universitatis Taurinensis (TO)
Source: Biodivers Data J. 2025 Feb 6;13:e134717. doi: 10.3897/BDJ.12.e134717 (PMC11826221; doi:10.3897/BDJ.12.e134717)
Supplement: Supplementary material 1 — Krona graph [file bdj-13-e134717-s001.html]

Javascript must be enabled to view this page.

magnitude

n. of taxa
n. of specimens


 630
 3424


 622
 3402


 8
 20


 8
 20


 3
 11


 2
 10


 1
 1


 2
 4


 2
 4


 1
 1


 1
 1


 2
 4


 1
 1


 1
 3


 3
 9


 3
 9


 3
 9


 3
 9


 10
 10


 2
 2


 1
 1


 1
 1


 1
 1


 1
 1


 1
 1


 1
 1


 1
 1


 3
 3


 3
 3


 2
 2


 1
 1


 4
 4


 4
 4


 4
 4


 33
 64


 4
 5


 4
 5


 1
 2


 1
 1


 1
 1


 1
 1


 1
 2


 1
 2


 1
 2


 28
 57


 28
 57


 2
 4


 1
 1


 6
 14


 1
 2


 1
 3


 1
 2


 1
 1


 1
 2


 2
 4


 2
 5


 3
 4


 6
 13


 1
 2


 1
 2


 1
 2


 1
 2


 1
 2


 562
 3287


 19
 77


 18
 75


 12
 57


 1
 1


 2
 6


 1
 1


 2
 10


 1
 2


 1
 2


 3
 5


 3
 5


 1
 1


 2
 4


 76
 307


 31
 65


 1
 1


 2
 13


 10
 12


 9
 19


 1
 7


 1
 2


 3
 4


 1
 2


 3
 5


 45
 242


 1
 7


 1
 1


 1
 6


 1
 1


 8
 25


 15
 112


 2
 5


 7
 46


 8
 38


 1
 1


 9
 140


 9
 140


 1
 34


 8
 106


 1
 7


 1
 7


 1
 7


 2
 4


 1
 3


 1
 3


 1
 1


 1
 1


 247
 1679


 5
 13


 3
 6


 2
 7


 43
 172


 43
 172


 2
 2


 2
 2


 54
 366


 1
 1


 7
 30


 28
 192


 6
 75


 2
 5


 4
 15


 4
 30


 1
 1


 1
 17


 89
 1002


 1
 3


 1
 1


 2
 30


 4
 12


 7
 93


 1
 1


 1
 6


 3
 49


 1
 37


 3
 71


 1
 1


 1
 5


 1
 10


 2
 4


 5
 50


 4
 31


 1
 2


 1
 2


 3
 12


 5
 95


 3
 35


 2
 70


 3
 10


 1
 1


 1
 2


 2
 26


 1
 5


 2
 85


 2
 14


 10
 54


 14
 185


 1
 6


 1
 6


 6
 20


 1
 1


 2
 7


 1
 1


 2
 11


 25
 48


 1
 2


 2
 2


 1
 1


 1
 1


 1
 2


 7
 12


 7
 23


 4
 4


 1
 1


 3
 4


 3
 4


 2
 2


 2
 2


 16
 40


 8
 20


 4
 10


 4
 10


 1
 4


 1
 4


 27
 146


 27
 146


 1
 7


 1
 1


 2
 2


 13
 75


 1
 8


 1
 1


 1
 1


 7
 51


 1
 1


 1
 1


 1
 1


 11
 36


 6
 28


 3
 18


 2
 9


 1
 1


 2
 2


 2
 2


 1
 4


 1
 4


 1
 1


 1
 1


 1
 1


 1
 1


 50
 193


 17
 32


 1
 3


 3
 11


 2
 3


 3
 3


 1
 1


 7
 11


 4
 20


 2
 18


 1
 1


 1
 1


 5
 7


 5
 7


 3
 6


 1
 2


 1
 3


 1
 1


 20
 126


 17
 107


 3
 19


 1
 2


 1
 2


 33
 152


 4
 14


 1
 4


 1
 1


 2
 9


 12
 96


 2
 16


 1
 1


 6
 69


 2
 9


 1
 1


 10
 29


 4
 17


 4
 7


 2
 5


 7
 13


 7
 13


 14
 173


 13
 159


 13
 159


 1
 14


 1
 14


 1
 5


 1
 5


 1
 5


 42
 187


 42
 187


 3
 9


 4
 19


 1
 3


 1
 1


 5
 25


 1
 1


 3
 4


 2
 4


 1
 1


 1
 2


 2
 10


 1
 1


 2
 27


 2
 21


 1
 1


 1
 1


 1
 1


 1
 1


 1
 3


 2
 8


 3
 25


 3
 19


 5
 9


 4
 4


 1
 1


 3
 3


 1
 5


 1
 5


 21
 166


 3
 5


 2
 3


 1
 2


 2
 21


 1
 19


 1
 2


 16
 140


 16
 140


 5
 10


 5
 10


 3
 8


 2
 6


 1
 2


 2
 2


 2
 2


 1
 1


 1
 1


 1
 1


 1
 1


 1
 1


 7
 21


 7
 21


 7
 21


 7
 21


 7
 21
